# Symplastic and apoplastic pathways for local distribution of silicon in rice leaves

**DOI:** 10.1111/nph.70110

**Published:** 2025-04-01

**Authors:** Sheng Huang, Naoki Yamaji, Noriyuki Konishi, Namiki Mitani‐Ueno, Jian Feng Ma

**Affiliations:** ^1^ Institute of Plant Science and Resources Okayama University Chuo 2‐20‐1 Kurashiki 710‐0046 Japan

**Keywords:** local distribution, mestome sheath, OsLsi6, rice, silicon, suberization, transporter, vascular bundle

## Abstract

- Silicon (Si) is highly accumulated in both the leaf blade and sheath of rice, but the transporter mediating the local distribution of Si between these two tissues remains unidentified.
- We investigated the role of an aquaporin, OsLsi6, in the local distribution of Si in rice leaves. We also examined the interrelations between vascular structure and OsLsi6 function in xylem unloading of Si for its local distribution.
- OsLsi6 is polarly localized at the xylem parenchyma cells of both the large and small vascular bundles of the leaf blade and sheath. OsLsi6 was downregulated by Si supply at the leaf sheath but not in the leaf blade. The knockout of *OsLsi6* increased the distribution of Si and germanium (Ge) to the leaf blade while reducing their distribution to the leaf sheath. The mestome sheath surrounding the vascular bundle was suberized in leaf sheaths and in large vascular bundles of leaf blades, but not in small vascular bundles of leaf blades.
- Our results indicate that there are two pathways for xylem unloading of Si for its local distribution: the OsLsi6‐dependent symplastic pathway in the leaf sheath and large vascular bundles of the leaf blade, and the apoplastic pathway in the small vascular bundle of the leaf blade.

## Introduction

Rice (*Oryza sativa*) is a typical accumulating plant of silicon (Si), which is able to accumulate Si in the shoots up to 10% of dry weight (Ma & Takahashi, 2002). This increase in accumulation is essential for high and stable production of rice (Tamai & Ma, 2008). Silicon is actively absorbed by the roots in the form of silicic acid, a noncharged molecule (Ma & Takahashi, 2002). After that, > 95% of Si absorbed is immediately translocated to the aboveground parts, including the leaf sheath and blade, and husk. In these tissues, silicic acid is polymerized to silica via transpiration, which is deposited beneath the cuticle of leaves and inside particular cells of leaf epidermis, forming silica cells and silica bodies or silica bulliform cells (motor cells; Ma & Takahashi, 2002). This deposition forms a mechanical barrier, which is important in protecting the plants from various stresses, such as pathogens, insect pests, drought, high salinity, metal toxicity, lodging, and nutrient imbalance stresses (Ma & Takahashi, 2002).

To transport Si from soil solution to different organs and tissues, different transporters involved in uptake, root‐to‐shoot translocation, and distribution, at least, are required. During the last two decades, transporters involved in different transport steps have been identified in rice (Huang & Ma, 2024). In terms of uptake, two transporters, including OsLsi1 and OsLsi2, have been identified. OsLsi1 belongs to the Nod26‐like major intrinsic protein (NIP) subfamily of aquaporin‐like proteins and functions as an influx transporter of Si (Ma *et al*., 2006), while OsLsi2 belongs to a putative anion transporter family without any similarity to OsLsi1 (Ma *et al*., 2007) and functions as an efflux transporter of Si. Both OsLsi1 and OsLsi2 are localized at the exodermis and endodermis in the mature root regions but show different polar localization. OsLsi1 is localized at the distal side, while OsLsi2 is localized at the proximal side (Ma *et al*., 2006; Yamaji & Ma, 2007), forming an efficient uptake system for Si (Huang & Ma, 2024). After uptake, Si as silicic acid is loaded into the root xylem by OsLsi3 (Huang *et al*., 2022), while it is unloaded from the xylem by OsLsi6 (Yamaji *et al*., 2008). OsLsi3, a homolog of OsLsi2, is localized at the root pericycle cells without polarity, while OsLsi6, a homolog of OsLsi1, is polarly localized at the adaxial side of the xylem parenchyma cells in leaf sheaths and leaf blades (Yamaji *et al*., 2008). Finally, the preferential distribution of Si to the husk is mediated by three different Si transporters: OsLsi6, OsLsi2, and OsLsi3, which are highly expressed in the nodes, especially in the node I (Yamaji & Ma, 2009; Yamaji *et al*., 2015). OsLsi6 is localized at the xylem transfer cells of enlarged vascular bundles, which is responsible for the unloading of Si from the xylem, while OsLsi2 and OsLsi3 are localized at the bundle sheath and parenchyma bridge cells, respectively, which are responsible for the further transfer of Si to the diffuse vascular bundles. Therefore, OsLsi6–OsLsi2–OsLsi3 localized at different cell layers of node I forms an efficient system of Si distribution (Yamaji & Ma, 2009; Yamaji *et al*., 2015; Huang & Ma, 2024). Recently, OsSIET4, a transporter belonging to the same family as OsLsi2 and OsLsi3, was found to be involved in Si deposition (Mitani‐Ueno *et al*., 2023). OsSIET4 is polarly localized at the distal side of epidermal cells and cells surrounding the bulliform cells of the leaf blade, where Si is deposited. The knockout of *OsSIET4* led to the death of rice grown in soil or nutrient solution containing Si but did not affect plant growth in the absence of Si, indicating its importance in exporting Si from leaf cells to the leaf surface for its proper deposition. However, although Si is highly accumulated in both the leaf sheath and the leaf blade, the transporter mediating local distribution of this element between two tissues remains unidentified.

On the other hand, it was reported that some metalloids share the same transporters for their uptake due to their similar chemical properties in terms of chemical species and molecular size (Yamaji & Ma, 2021). For example, rice OsLsi1/OsNIP2;1 shows transport activity for arsenite (As(OH)_3_), boric acid (B(OH)_3_), selenite (Se(OH)_3_), silicic acid (Si(OH)_4_), and antimonite (Sb(III)) (Ma *et al*., 2006, 2008; F. J. Zhao *et al*., 2010; X. Q. Zhao *et al*., 2010; Pommerrenig *et al*., 2015; Shao *et al*., 2018; Yamaji & Ma, 2021; H. Huang *et al*., 2024). However, whether these metalloids share the same pathway for their local distribution between the leaf blade and the leaf sheath in rice remains unknown. In the present study, through a detailed analysis, we found that OsLsi6 is localized at both large and small vascular bundles of the leaf blade and sheath and that it is involved in xylem unloading for the local distribution of Si, as well as Ge (in the form of germanic acid), an analog of Si, between the leaf sheath and the leaf blade in rice. By contrast, the contribution of OsLsi6 to the local distribution of boron (B) and arsenic (As) is negligible. Furthermore, by investigating and comparing different vascular structures of large and small vascular bundles in the leaf sheath and blade, we revealed a symplastic pathway, which is mediated by OsLsi6, and an OsLsi6‐independent apoplastic pathway for the local distribution of Si and Ge in rice.

## Materials and Methods

### Plant materials and growth condition

Wild‐type (WT) rice (*Oryza sativa* L. cv Nipponbare) and two independent knockout lines of *OsLsi6* generated as described below were used. Seeds were soaked in water overnight at 30°C in the dark for 2 d and then transferred onto a net floating on a solution containing 0.5 mM CaCl_2_. On Day 7, seedlings were transferred to a 3.5‐l plastic pot containing one‐half strength Kimura B solution (pH 5.6; Ma *et al*., 2002). The nutrient solution was renewed every 2 d. The plants were grown in a controlled glasshouse at 25–30°C, under natural light and used for the following analysis. All experiments were performed with three to four biological replicates.

### Generation of *oslsi6* knockout lines by CRISPR/Cas9

The knockout lines of *OsLsi6* were generated by the CRISPR/Cas9 technique. Twenty bases upstream of the protospacer adjacent motif were selected as candidate target sequences (Supporting Information Fig. S1). The primers for two target sequences at the first exon and the second exon are listed in Table S1. The single guide RNA vector (pU6gRNA) and plant expression vector of Cas9 (pZDgRNA_Cas9VER.2_HPT) were used as described previously (Mikami *et al*., 2015). The derived construct was transformed into rice (cv Nipponbare) calluses according to Hiei *et al*. (1994).

To genotype the resultant mutants, genomic DNA was extracted from leaves of transgenic lines. Polymerase chain reaction (PCR) amplifications were carried out using primer pairs flanking the designed target sites as listed in Table S1. PCR products (*c*. 450 bp) were sequenced directly using internal specific primers, of which the binding positions are desirably at *c*. 200‐bp upstream of the target sites. Two homologous knockout lines (*lsi6 A‐2* and *lsi6 B‐29*) without Cas9 were selected, and the T3 generation lines were used in subsequent phenotypic analysis.

### Leaf sheath‐ and blade‐dependent expression analysis of *OsLsi6* and its response to Si

The seedlings (14‐d‐old) were cultivated in a nutrient solution in the absence or presence of 1 mM Si as silicic acid. Silicic acid was prepared by passing potassium silicate through cation‐exchange resin (Amberlite IR‐120B, H^+^ form; Organo, Tokyo, Japan; Ma *et al*., 2001). After 1 wk, the leaf sheath and the blade of Leaves 5 and 6 (fully expanded leaves) and Leaf 7 (unexpanded newest leaf) were sampled for RNA extraction. The leaf sheath and the blade of Leaf 5 were also sampled for immunostaining as described below. For expression analysis, the total RNA from different tissues was extracted using an RNeasy Plant Mini Kit (Qiagen). cDNA was synthesized by ReverTra Ace qPCR RT Kit (Toyobo, Osaka, Japan) according to the manufacturer's instruction. The expression analysis of *OsLsi6* was determined with SsoAdvanced Universal SYBR Green Supermix (Bio‐Rad) on a real‐time PCR machine CFX384 (Bio‐Rad). Both *Histone H3* and *Actin* were used as internal controls. Relative expression levels were calculated by the ΔΔ*C*_t_ method. Four independent biological replicates were performed for each treatment. The primer sequences used are shown in Table S1.

### Immunostaining of OsLsi6 protein

Immunostaining was performed using a specific antibody against OsLsi6 reported in a previous study (Yamaji *et al*., 2008). For the observation of OsLsi6 localization, the leaf blade and the leaf sheath of Leaf 7 (fully expanded newest leaf) were fixed in 4% (w/v) paraformaldehyde and 60 mM Suc buffered with 50 mM cacodylic acid (pH 7.4) for 2 h at room temperature with occasional degassing. After washing three times by PBS, the fixed samples were embedded in 5% agar and sectioned 100‐mm thick with a micro‐slicer (ZERO 1; Dosaka EM, Kyoto, Japan; Yamaji *et al*., 2008). To investigate the response to Si supply, the leaf blade and sheath of Leaf 5 with and without Si for 1 wk were used as described above. Fluorescence from secondary antibodies (Alexa Fluor 555 goat anti‐rabbit IgG; Thermo Fisher Scientific, Waltham, MA, USA) was observed using a confocal laser scanning microscopy (TCS SP8x; Leica Microsystems, Wetzlar, Germany). For quantification of protein level, the signal intensity was quantified using the line quantification tool of the Las Af Lite software, v.4.0 (Leica Microsystems). Nine independent slices from three independent plants (3 × 3) were used for quantification.

### Suberin staining in leaf

Leaf 5 of WT rice (24‐d‐old) grown hydroponically was used for suberin staining. Cross sections of the leaf blade and the leaf sheath with 100 μm thickness were prepared by microslicer (ZERO1; Dosaka EM). Suberin staining was performed with fluorol yellow 088 fluorescent dye according to Lux *et al*. (2005). Fluorescence was observed with a confocal laser scanning microscopy (TCS SP8x).

### Apoplastic dye tracer experiment

For the apoplastic permeability experiment, trisodium‐8‐hydroxy‐1,3,6‐pyrenetrisulphonic acid (PTS) as a fluorescent apoplastic dye was used (Peterson *et al*., 1985; Faiyue *et al*., 2010). Half roots of the WT rice (21‐d‐old) grown hydroponically at the five‐leaf stage were cut by a sharp razor blade, and then, the remaining parts, including the shoot, were exposed to a solution containing 0 or 0.2 mM PTS at room temperature. After 1 h, the middle parts of the fully expanded fourth leaf blade and leaf sheath were cut *c*. 2 cm long and mounted on a slide using tape to prevent leaf curling. Fluorescence was observed immediately after the sample was prepared, with a confocal laser scanning microscopy (TCS SP8x; Leica Microsystems).

### Determination of transport activity of OsLsi6 for boric acid

Transport activity of OsLsi6 for boric acid was performed by the swelling assay using *Xenopus laevis* oocytes. Procedures for deflocculation, culture conditions, and selection were the same as described previously (Ma *et al*., 2006). cRNA of *OsLsi6* was synthesized *in vitro* (Mitani *et al*., 2008). Oocytes injected with water (negative control), the cRNA of *OsLsi6* or *OsLsi1* as a positive control, were incubated in the MBS buffer (88.0 mM NaCl, 1.0 mM KCl, 2.4 mM NaHCO_3_, 15.0 mM Tris–HCl, pH 7.6, 0.3 mM Ca(NO_3_)_2_, 0.41 mM CaCl_2_, 0.82 mM MgSO_4_, 10 μg ml^−1^ sodium penicillin, and 10 μg ml^−1^ streptomycin sulfate) at 18°C. After 2 d of incubation, oocytes were transferred to an isotonic solution containing one‐fifth of diluted MBS supplemented with boric acid to adjust the osmolarity (boric acid concentration was 175 mM). Changes in the oocyte volume were monitored within 180 s at 20 s intervals. Permeability of boric acid was presented as oocyte volume change (V/V_0_).

### Phenotypic analysis of *OsLsi6* mutants in Si allocation

Seedlings (10‐d‐old) of both WT and two *oslsi6* mutants were grown in a solution containing 1 mM Si (+Si) as silicic acid for 12 d. The solution was changed once every 2 d. At harvest, the leaf blade and the leaf sheath of Leaves 5 and 6 (fully expanded leaves) and Leaf 7 (unexpanded newest leaf) were separately sampled and subjected to the determination of Si and other elements as described below.

### Short‐term labeling experiments with Ge, ^10^B, and As

For a short‐term labeling experiment with Ge or As, seedlings (20‐d‐old) of both WT and two mutants were exposed to a solution containing 2 μM Ge or 2 μM arsenite in the presence of 1 μM of rubidium (Rb) and strontium (Sr). Since Rb and Sr show similar chemical properties to potassium (K) and calcium (Ca), respectively, they have been used as symplastic tracers and apoplastic tracers (Kuppelwieser & Feller, 1991). After a 2‐d exposure, the roots were washed three times with 5 mM cold CaCl_2_ and separated from the shoots. The shoot part was subsequently separated into the shoot basal region (5 mm from the root‐to‐shoot junction), old leaves (Leaves 2–4), leaf sheath and leaf blade of Leaves 5 and 6 (fully expanded leaves), and Leaf 7 (unexpanded newest leaf) and subjected to the determination of mineral element concentration as described below.

For a short‐term labeling experiment with ^10^B, the seedlings (20‐d‐old) grown in 3 μM ^11^B were exposed to a solution containing 3 μM ^10^B. After 2 d, the plants were subsequently separated into root, shoot basal region (5 mm from the root‐to‐shoot junction), old leaves (Leaves 2–4), leaf sheath and leaf blade of Leaves 5 and 6 (fully expanded leaf), and Leaf 7 (unexpanded newest leaf). All samples harvested were subjected to the determination of mineral element concentration as described below. The distribution ratio in each organ is calculated by (content in each organ/total content) × 100%.

### Determination of Si and other mineral element concentration

All samples harvested were dried at 70°C in an oven for at least 2 d. For Si determination, the sample was microwave‐digested in a mixture of 3 ml of HNO_3_ (61%), 3 ml of hydrogen peroxide (30%), and 2 ml of hydrofluoric acid (46%) (Microwave Digestion System START D; Millstone Co. Ltd, Tuscany, Italy), and the digested sample was diluted to 50 ml with 4% boric acid. The Si concentration in the digest solution was determined by the colorimetric molybdenum blue method at 600 nm as described previously (Okuda & Takahashi, 1961).

For the determination of other mineral elements, the dried samples were digested with concentrated HNO_3_ (61%) at a temperature up to 135°C till the solution became clear. The concentration of mineral elements in the digested solution was determined by ICP‐MS (7700X; Agilent Technologies, Santa Clara, CA, USA). For the determination of ^11^B and ^10^B, isotope mode was employed; ^11^B and ^10^B were measured separately and distinguished based on their mass‐to‐charge ratio (*m/z*).

### Statistical analysis

Statistical comparison was performed by one‐way ANOVA followed by Tukey's test. The significance of differences was defined as *P* < 0.05 or *P* < 0.01.

## Results

### Localization of OsLsi6 at large and small vascular bundles in leaf blade and leaf sheath

**Fig. 1:**
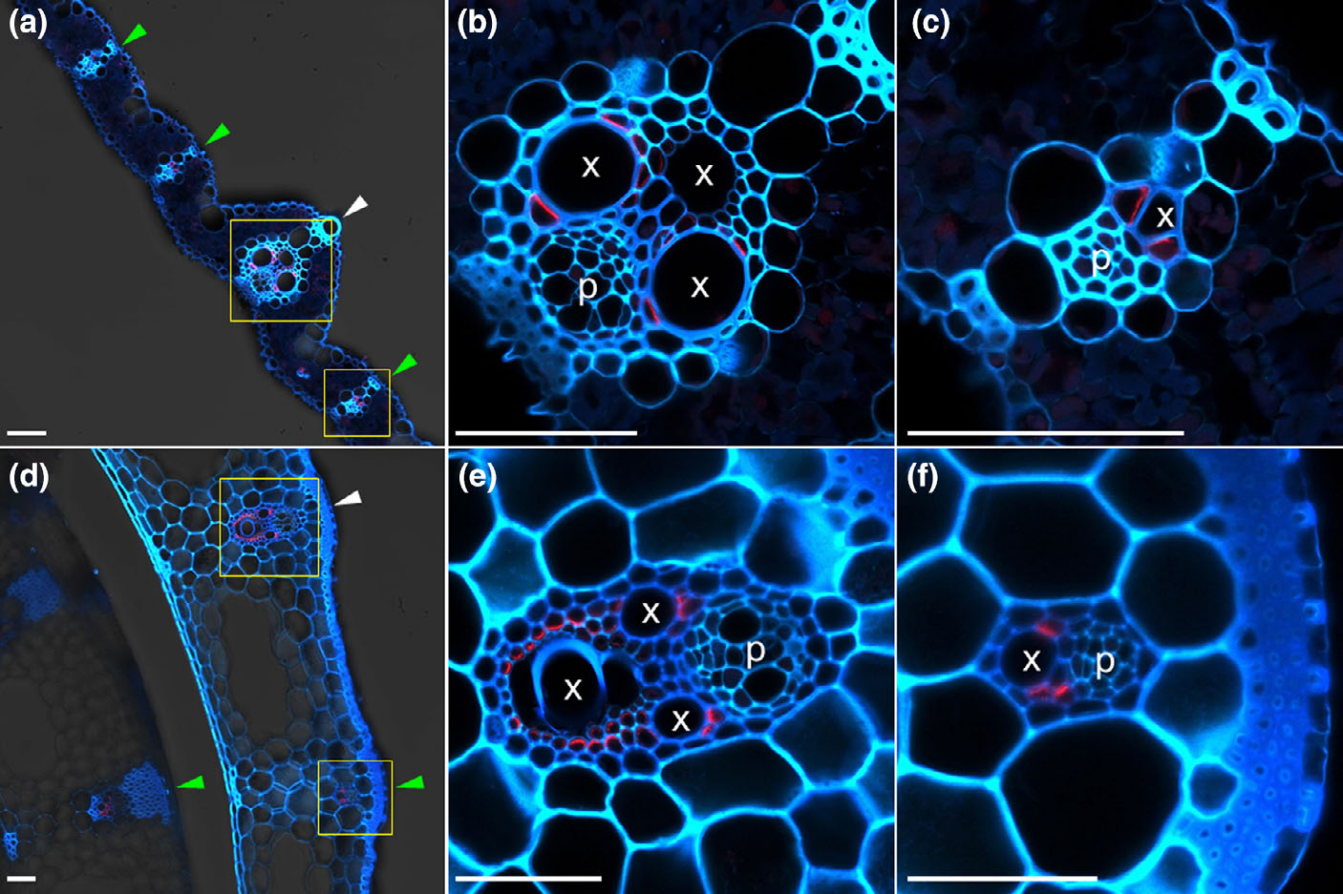
Tissue‐ and cell‐specific localization of OsLsi6 protein in the leaf blade and the leaf sheath of rice. (a–c) Localization of OsLsi6 protein in the leaf blade. (d–f) Localization of OsLsi6 protein in the leaf sheath. (b, c, e, f) Close‐up image of a large vascular bundle (b, e) and a small vascular bundle (c, f) in the leaf blade (b, c) and the leaf sheath (e, f) are shown. Immunostaining with OsLsi6 antibody was performed using the seventh fully expanded leaf. Red color indicates signal from OsLsi6, and blue and cyan colors from autofluorescence of the cell wall. White and green arrowheads in (a, d) indicate the large and small vascular bundles, respectively. p, phloem; x, xylem vessel. Bars, 50 μm.

By immunostaining using a specific antibody against OsLsi6 (Yamaji *et al*., 2008), we further observed its tissue‐specific localization in both the leaf blade and the leaf sheath of rice. In addition to the localization at the xylem parenchyma cells of the large vascular bundle, which was similar to that observed previously (Yamaji *et al*., 2008), the signal was also detected in the xylem parenchyma cells of the small vascular bundles of both the leaf blade and the leaf sheath (Fig. 1a,d). Furthermore, in both large and small vascular bundles, OsLsi6 was polarly localized at the side facing toward the xylem vessel (Fig. 1b,c,e,f).

**Fig. 2:**
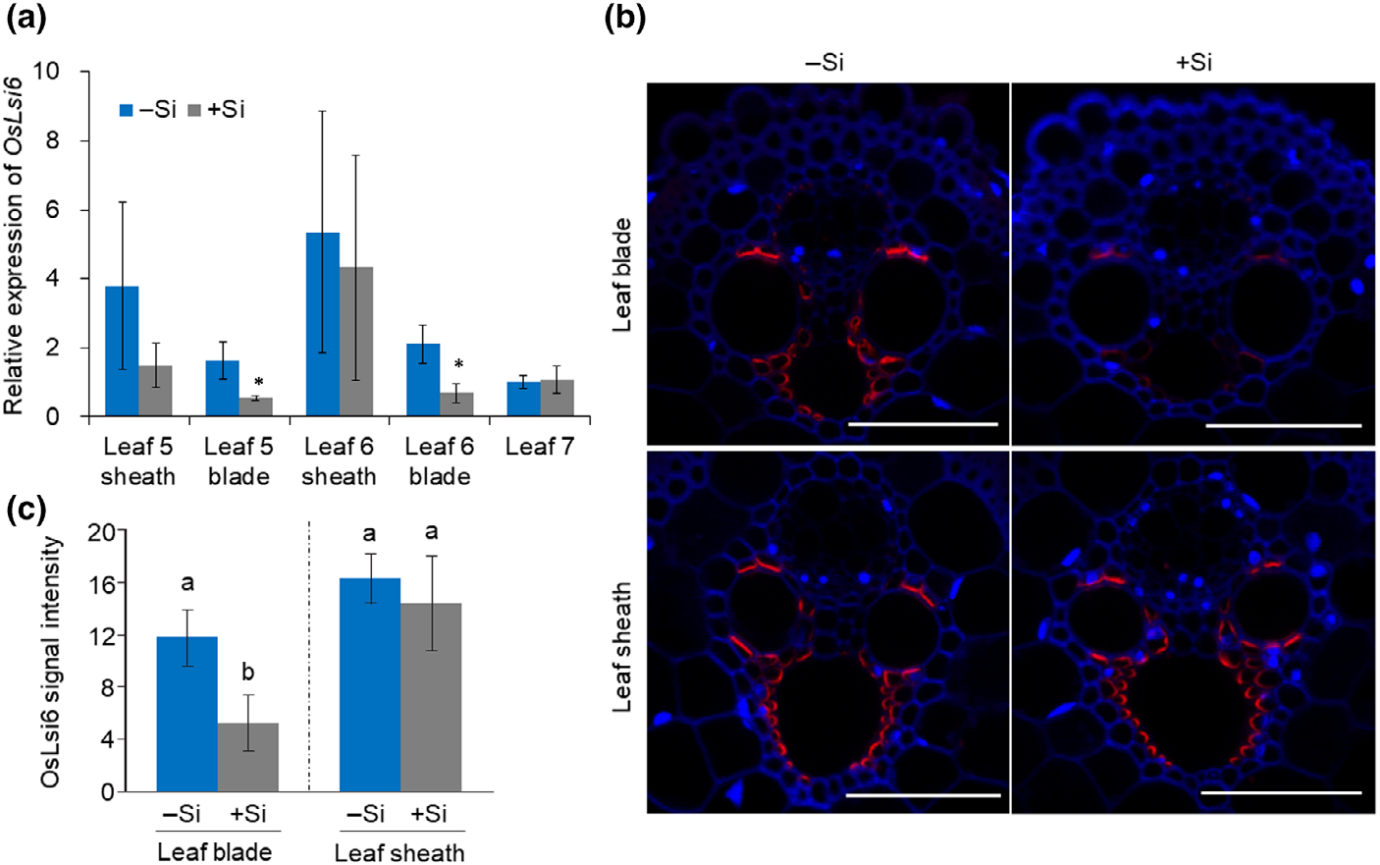
Response of OsLsi6 to silicon (Si) supply in the leaf sheath and the leaf blade of rice. (a) Tissue‐dependent expression of *OsLsi6* gene in response to Si supply. Seedlings were exposed to a solution containing 0 or 1 mM Si for 7 d. Different tissues, including the leaf blade and the leaf sheath of Leaves 5 and 6 (fully expanded leaves) and Leaf 7 (unexpanded newest leaf), were separately harvested for RNA extraction. Expression of *OsLsi6* was determined by quantitative reverse transcription polymerase chain reaction. Both *Histone H3* and *Actin1* were used as internal controls. The expression relative to Leaf 7 (−Si) is shown. Data are means ± SD (*n* = 4). Significant difference compares with −Si condition was marked with *, *P* < 0.05 by ANOVA followed by Tukey's test. (b) Response of OsLsi6 protein to Si supply in the leaf blade and the leaf sheath. The fifth leaf (fully expanded) with or without Si supply for 7 d was used for immunostaining with OsLsi6 antibody. Red color indicates signal from OsLsi6, and blue color from autofluorescence of the cell wall. Bars, 50 μm. (c) Signal intensity of OsLsi6 in the large vascular bundle of the leaf blade and the leaf sheath. Data are means ± SD of nine different slices from three independent plants. Different letters indicate significant differences by ANOVA followed by Tukey–Kramer's test (*P* < 0.05).

To investigate the response of *OsLsi6* expressed in the leaf blade and sheath to Si supply, we first compared its expression level in different leaves between plants with and without Si supply. The expression of *OsLsi6* in the leaf sheath was not altered by Si supply irrespective of leaf position, whereas in the leaf blade it was downregulated by Si in both Leaves 5 and 6 (Fig. 2a). Consistent with this result, the OsLsi6 protein in the leaf sheath of Leaf 5 was not changed by Si supply, but in the leaf blade it was significantly decreased (Fig. 2b,c).

### Si accumulation in leaf sheath and blade

**Fig. 3:**
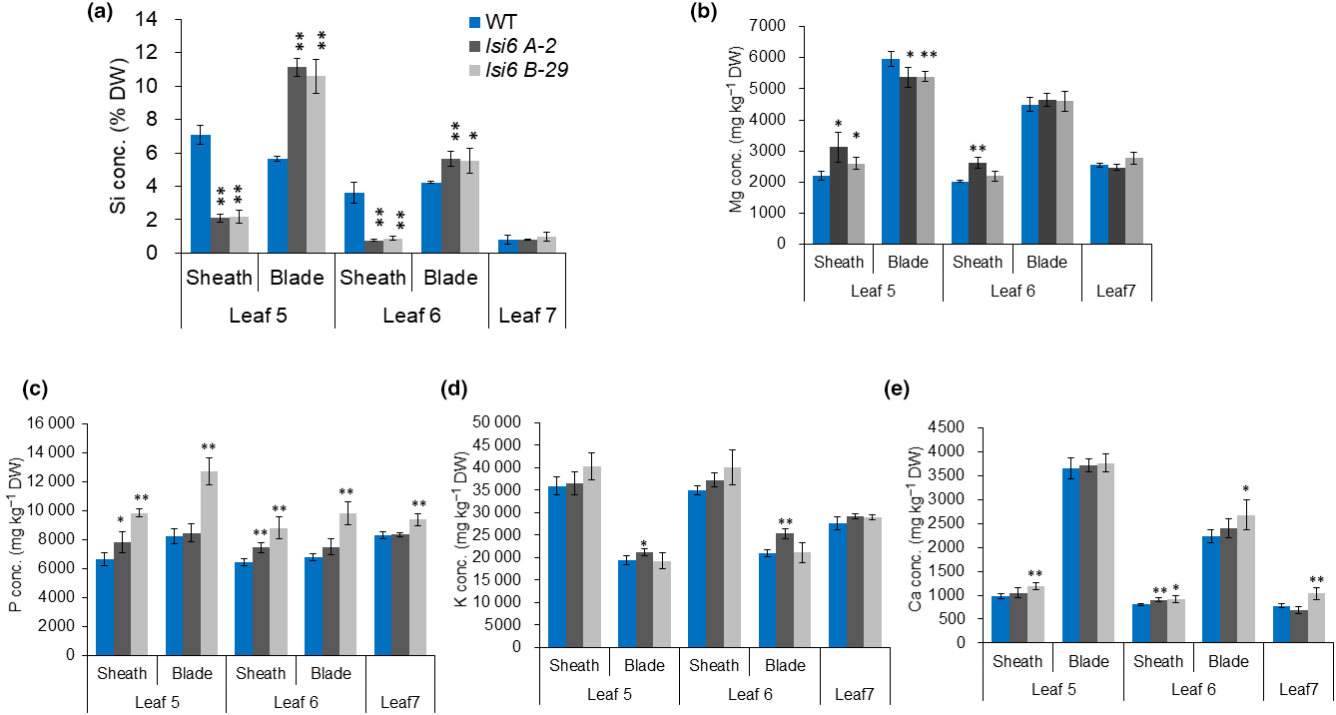
Tissue‐dependent accumulation of silicon (Si) in the rice leaf blade and sheath of wild‐type (WT) rice and two knockout lines of OsLsi6. (a–e) Concentration of Si (a), magnesium (Mg) (b), phosphorus (P) (c), potassium (K) (d), and calcium (Ca) (e) in the leaf sheath and the leaf blade of Leaves 5 and 6 (fully expanded leaves) and Leaf 7 (unexpanded newest leaf). Seedlings of both WT and mutants were grown hydroponically in the presence of 1.0 mM Si for 12 d. The leaf blade and sheath were separately harvested and subjected to the determination of Si and other elements. Data are means ± SD (*n* = 4). Asterisks above bars indicate significant differences compared with WT (*, *P* < 0.05; **, *P* < 0.01), as determined by ANOVA followed by Tukey's test.

Based on the tissue‐specificity of OsLsi6 localization (Fig. 1), we speculated that it is involved in xylem unloading of Si in the leaf sheath and blade. We, therefore, compared Si accumulation in the leaf sheath and blade of different leaves between the WT rice and two independent knockout lines of *OsLsi6*. The knockout lines were generated by the CRISPR/Cas9 technique using two different target sites (Fig. S1). *lsi6 A‐2* has a 2‐bp deletion at the first exon, while *lsi6 B‐29* has a 1‐bp insertion at the second exon. We grew these lines in a hydroponic solution containing 1.0 mM Si for 12 d and then determined the Si concentration in the leaf blade and the leaf sheath of Leaves 5 and 6 (fully expanded leaves) and Leaf 7 (not expanded new leaf). The Si concentration in the leaf sheath was significantly lower in both Leaves 5 and 6 of the mutants than that in the WT (Fig. 3a), whereas in the leaf blade it was higher in the mutants than in the WT. The Si concentration in the unexpanded leaf (Leaf 7) was much lower compared with expanded leaves (Leaves 5 and 6), and no difference in the Si concentration was observed between WT and mutants (Fig. 3a). We also compared other element profiles between WT and mutants, but no consistent differences were observed (Figs 3b–e, S2). One of the knockout lines showed high accumulation of phosphorus (P) due to unknown reasons. These results indicate that the knockout of *OsLsi6* altered the local distribution of Si between the leaf sheath and the leaf blade.

### Short‐term labeling experiment with Ge

**Fig. 4:**
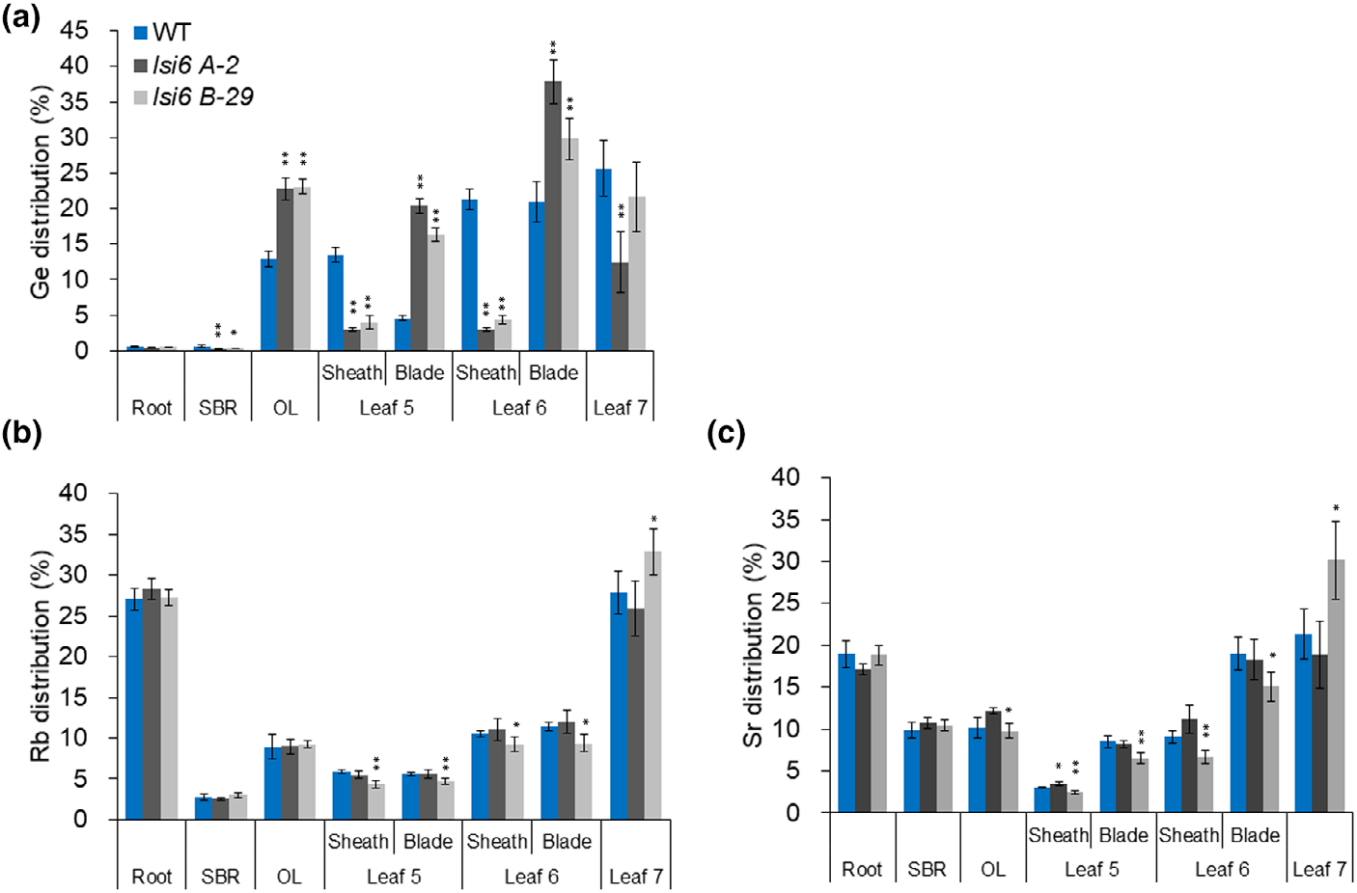
Effect of *OsLsi6* knockout on local distribution of germanium (Ge) in rice leaf. (a–c) Distribution of Ge (a), Rb (b) and Sr (c) in different tissues of wild‐type (WT) rice and two independent knockout lines of *OsLsi6*. Seedlings (20‐d‐old) were exposed to a solution containing 2 μM Ge in the presence of 1 μM of Rb and Sr for 2 d. Different organs and tissues were separately harvested as shown in the figure and subjected to the determination of Ge, Rb, and Sr. OL, old leaves (Leaves 2–4); SBR, shoot basal region. Data are means ± SD (*n* = 4). Asterisks above bars indicate significant differences compared with WT (*, *P* < 0.05; **, *P* < 0.01), as determined by ANOVA followed by Tukey's test.

To confirm the above results, we performed a short‐term (2 d) labeling experiment with Ge. Germanium is an analog of Si and shows similar transport behavior as Si in plants (Takahashi *et al*., 1976). The knockout of *OsLsi6* resulted in decreased Ge accumulation in the leaf sheath of Leaves 5 and 6 (fully expanded leaves), but increased accumulation in the leaf blade of these leaves (Fig. 4a). A higher Ge concentration was also found in old leaves (Leaves 2–4) of knockout lines compared with the WT (Fig. 4a), but the difference in Ge accumulation in Leaf 7 (unexpanded leaf) was not significant. By contrast, no difference was found in the accumulation of Rb and Sr in the leaf sheath and blade between WT and knockout lines (Fig. 4b,c), which were used as a control of the symplastic and apoplastic tracers, respectively. These results are consistent with Si accumulation (Fig. 3a), supporting that the knockout of *OsLsi6* altered the local distribution of Si and Ge between the leaf sheath and blade.

### Contribution of OsLsi6 to local distribution of As and B

**Fig. 5:**
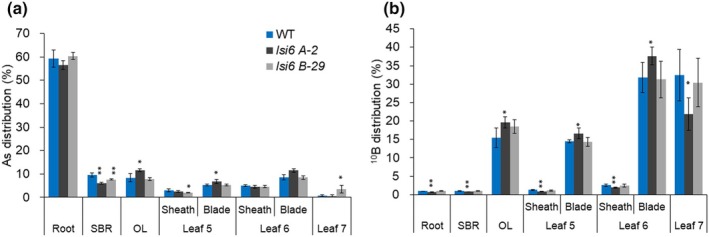
Effect of *OsLsi6* knockout on local distribution of arsenic (As) and boron (B) in rice leaf. (a, b) Distribution of As (a) and ^10^B (b) in different tissues of wild‐type (WT) rice and two independent knockout lines of *OsLsi6*. Seedlings (20‐d‐old) were exposed to a solution containing 2 μM As (as arsenite) for 2 d. For ^10^B labeling experiment, seedlings (20‐d‐old) hydroponically grown in ^11^B were exposed to a solution containing 3 μM ^10^B for 2 d. After the exposure, different organs and tissues were separately harvested as shown in the figure and subjected to determination of As and ^10^B. OL, old leaves (Leaves 2–4); SBR, shoot basal region. Data are means ± SD (*n* = 4). Asterisks above bars indicate significant differences compared with WT (*, *P* < 0.05; **, *P* < 0.01), as determined by ANOVA followed by Tukey's test.

Since OsLsi6 is also permeable to As(III) (Ma *et al*., 2008), its contribution to the local distribution of As was investigated. After a 2‐d exposure to arsenite, unlike Ge (Fig. 4a), *c*. 60% of total As was accumulated in the roots (Fig. 5a). No significant differences in As accumulation in both the leaf blade and the leaf sheath of different leaves were found (Fig. 5a), indicating that the contribution of OsLsi6 to the local distribution of As is negligible.

We also examined the contribution of OsLsi6 to the local distribution of B. First, we determined the transport activity of OsLsi6 for boric acid in *Xenopus* oocytes using OsLsi1 as a positive control (Shao *et al*., 2018). Similar to OsLsi1, OsLsi6 was also permeable to boric acid (Fig. S3). We then grew the plants with ^11^B, followed by growing in a solution labeled with ^10^B for 2 d. Most ^10^B was distributed to old leaves (Leaves 2–4), the leaf blade of Leaves 5 and 6, and Leaf 7 in both WT and knockout lines (Fig. 5b), whereas only a small percentage of total ^10^B was distributed to the leaf sheath. A slight difference in ^10^B distribution to the leaf sheath and blade was observed between WT and mutants (Fig. 5b), suggesting that OsLsi6 plays only a minor role in the local distribution of B in rice.

### Suberization of mestome sheath in leaf blade and leaf sheath

**Fig. 6:**
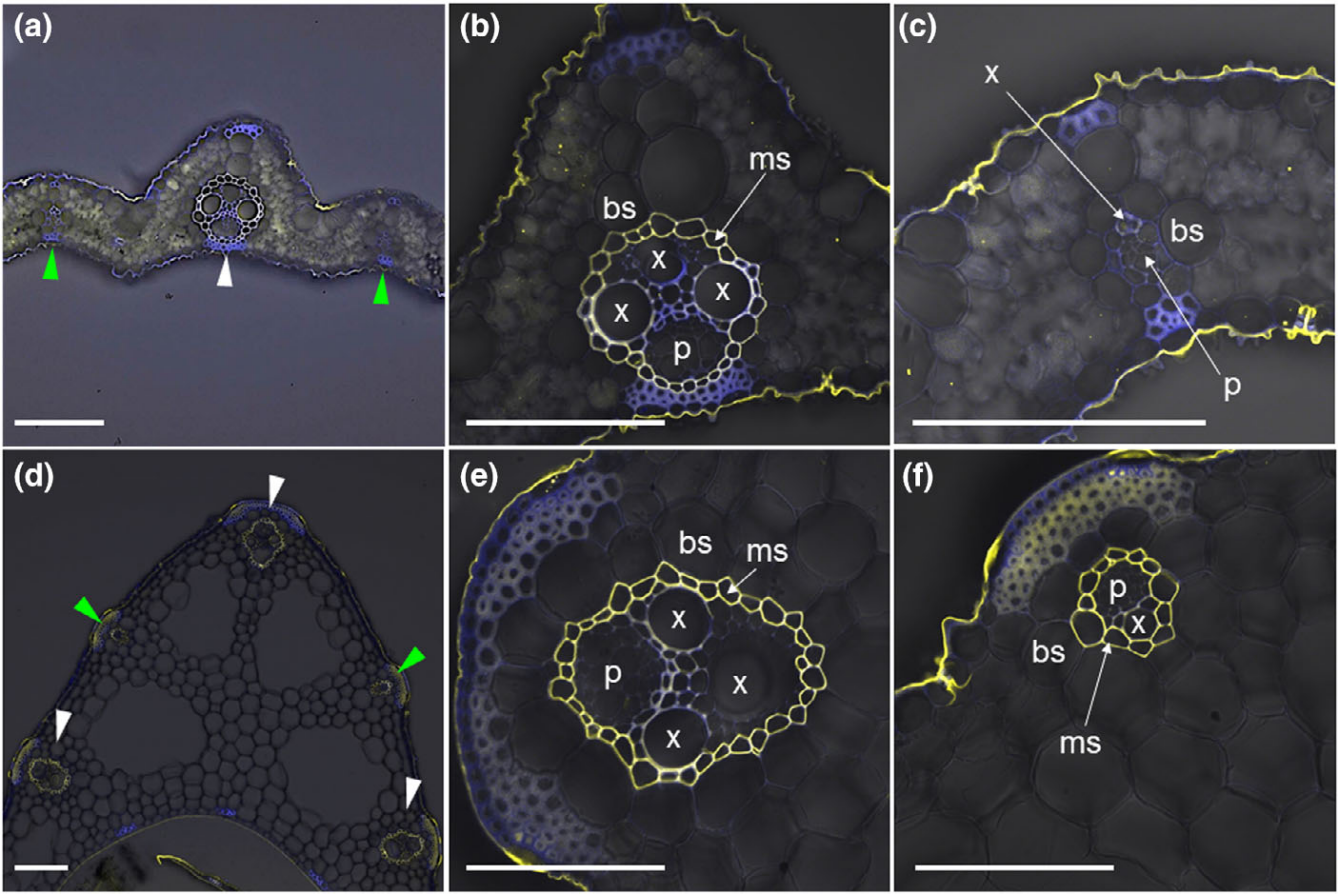
Suberin staining in the leaf blade and the leaf sheath of rice. (a–c) Suberin staining in the leaf blade. (d–f) Suberin staining in the leaf sheath. (b, c, e, f) Magnified images of a large vascular bundle (b, e) and a small vascular bundle (c, f) in the leaf blade (b, c) and the leaf sheath (e, f) are shown. Leaf cross sections from the fourth fully expanded leaf were prepared and stained by fluorol yellow 088. Yellow color shows suberin, and blue color is from cell wall autofluorescence. White and green arrowheads indicate the large and small vascular bundles, respectively, in (a, d). bs, bundle sheath; ms, mestome sheath; p, phloem; x, xylem vessel. Bars, 100 μm.

To link the local distribution of metalloids with vascular structure, we investigated the suberin deposition at the mestome sheath of vascular bundles by staining with a fluorescent dye, fluorol yellow 088. In large vascular bundles of both the leaf blade and sheath, we observed the mestome sheath heavily suberized surrounding the vasculature (Fig. 6a,b,d,e). The suberized mestome sheath was also observed at the small vascular bundle of the leaf sheath (Fig. 6f), but small vascular bundles of the leaf blade were not suberized (Fig. 6c). These results were consistent with previous studies in rice leaf (Kaneko *et al*., 1980; Chonan *et al*., 1981, 1984, 1985).

### Apoplastic permeability from vascular bundles in leaf blade and leaf sheath

**Fig. 7:**
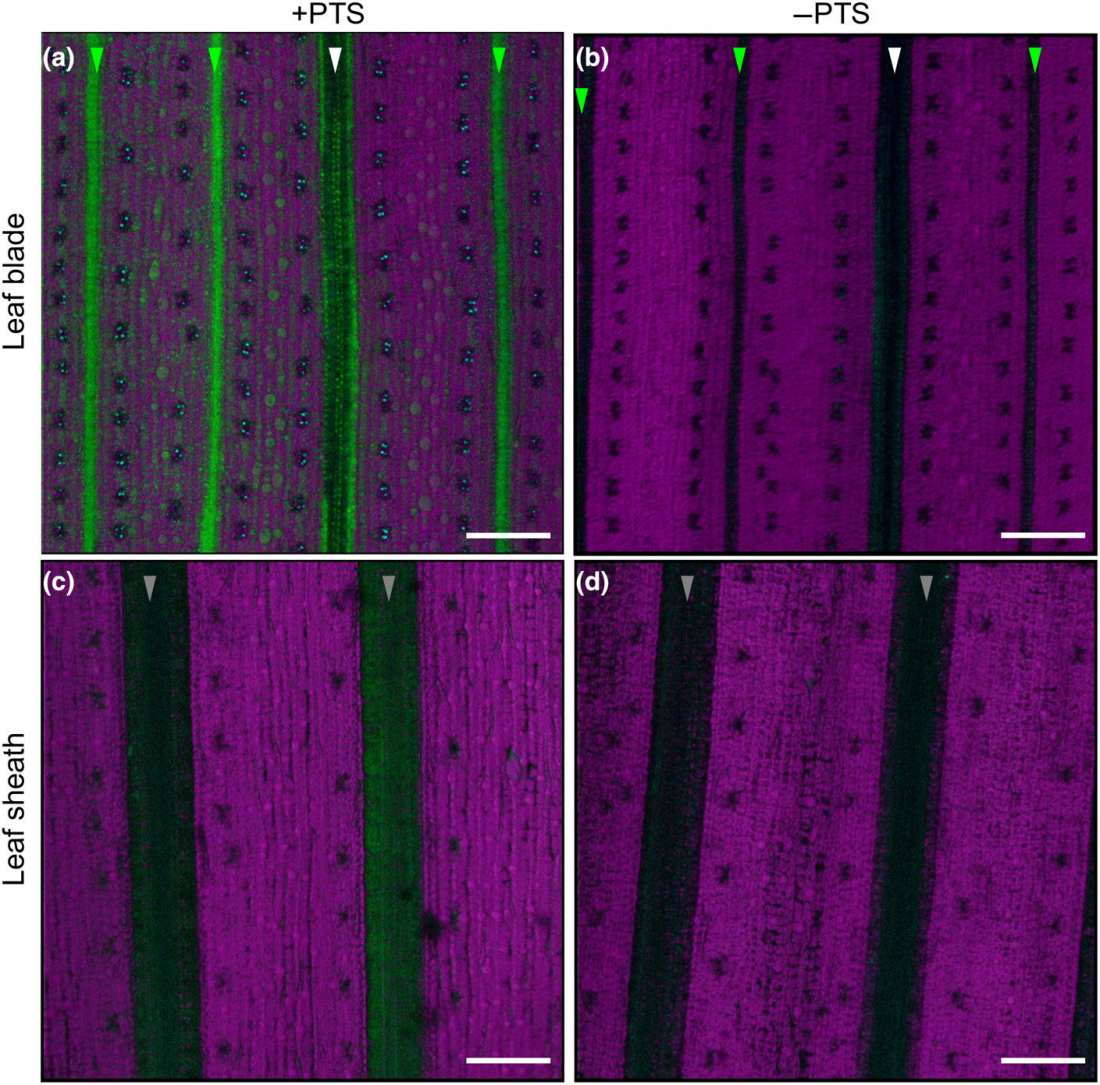
Apoplastic permeability test of the leaf blade and the leaf sheath of rice. (a, b) Fluorescence of trisodium‐8‐hydroxy‐1,3,6‐pyrenetrisulphonic acid (PTS) in the leaf blade. (c, d) Fluorescence of PTS in the leaf sheath. The half roots of the seedlings (21‐d‐old) were cut and exposed to an aqueous solution with (a, c) or without (b, d) 0.2 mM PTS. After 1 h, the fluorescence in the leaf blade and the leaf sheath abaxial side of a fully expanded youngest leaf was observed immediately under a microscope. Green color shows fluorescence of PTS (green) and magenta color is Chl autofluorescence. White, green, and gray arrowheads indicate veins along with the large (white) and small (green) vascular bundles in the leaf blade and in the leaf sheath (gray; unrecognized large‐ or small‐), respectively. Bars, 100 μm (a–d).

To investigate the effect of suberization of the mestome sheath on the apoplastic pathway, we fed fluorescent apoplastic dye (PTS) from the root for 1 h. To avoid the effects of Casparian strips in the roots, half of the roots was cut before PTS exposure. On the abaxial side of the leaf blade, strong PTS fluorescence was observed, especially around the leaf vein along with small vascular bundles of plants fed with PTS (Fig. 7a), but no signal was observed in the plants without PTS feeding (Fig. 7b). By contrast, only trace fluorescence was observed at the leaf vein of the leaf sheath with PTS feeding (Fig. 7c) and no signal was observed in the leaf sheath without PTS feeding (Fig. 7d). These observations indicate that the xylem sap is leaked from vascular bundles in the leaf blade, whereas it is fully blocked in the leaf sheath due to mestome suberization.

## Discussion

After being taken up by the roots, followed by loading to the xylem vessels, the mineral elements will be translocated to the aboveground parts through the transpiration stream. During the translocation process, mineral elements must be unloaded from the xylem vessel to different leaf tissues for their growth requirements. This process is required for both long‐distance transport and local distribution. However, compared with uptake and xylem loading, less is understood about the molecular mechanisms underlying xylem unloading. Recently, OsBOR1 and OsNramp5 in rice were reported to be involved in xylem unloading for B and Mn, respectively (Shao *et al*., 2021; S. Huang *et al*., 2024). They are localized at the xylem parenchyma cells of the leaf sheath and are responsible for the local distribution. In the present study, through further detailed functional characterization of OsLsi6 combined with structural analysis, we revealed two different pathways for the local distribution of Si as well as Ge in rice.

### OsLsi6 is involved in local distribution of Si, but not B and As

In the present study, we investigated the role of OsLsi6 in xylem unloading for local distribution of Si. We also compared the other two metalloids, B and As, with Si in terms of the local distribution of these elements. These metalloids are similarly present in the form of noncharged molecules, B(OH)_3_, Si(OH)_4_, and As(OH)_3_, due to their similar p*K*_a_ (Yamaji & Ma, 2021). In fact, OsLsi6 shows transport activity for these metalloids in a heterologous assay system (Ma *et al*., 2008; Yamaji *et al*., 2008; Fig. S3). However, we found that the role of OsLsi6 in xylem unloading differs among these metalloids. OsLsi6 plays an important role in xylem unloading of Si and Ge, whereas its role in xylem loading of As and B is negligible. This is supported by the findings that the knockout of *OsLsi6* significantly altered the distribution of Si and Ge between the leaf sheath and the leaf blade (Figs 3a, 4a), whereas its knockout hardly affected the distribution of As and B (Fig. 5a,b). These differences could be attributed to their different distribution patterns within the plants. Labeling experiments showed that > 90% of Ge and B taken up by the roots was translocated to the shoots (Figs 4a, 5b), whereas 60% of As taken up was retained in the roots (Fig. 5a). Within the shoots, more Ge was distributed to the leaf sheath (Fig. 4a); by contrast, B was distributed more to the leaf blade (Fig. 5b). Since OsNIP3;1 was reported to show similar tissue localization as OsLsi6 in the leaf sheath and blade (Shao *et al*., 2018), there is a possibility that the local distribution of B is mediated by OsNIP3;1. These results suggest that different transporters are involved in xylem unloading of these metalloids for their local distribution.

### Symplastic and apoplastic pathways for local distribution of Si in rice leaves

**Fig. 8:**
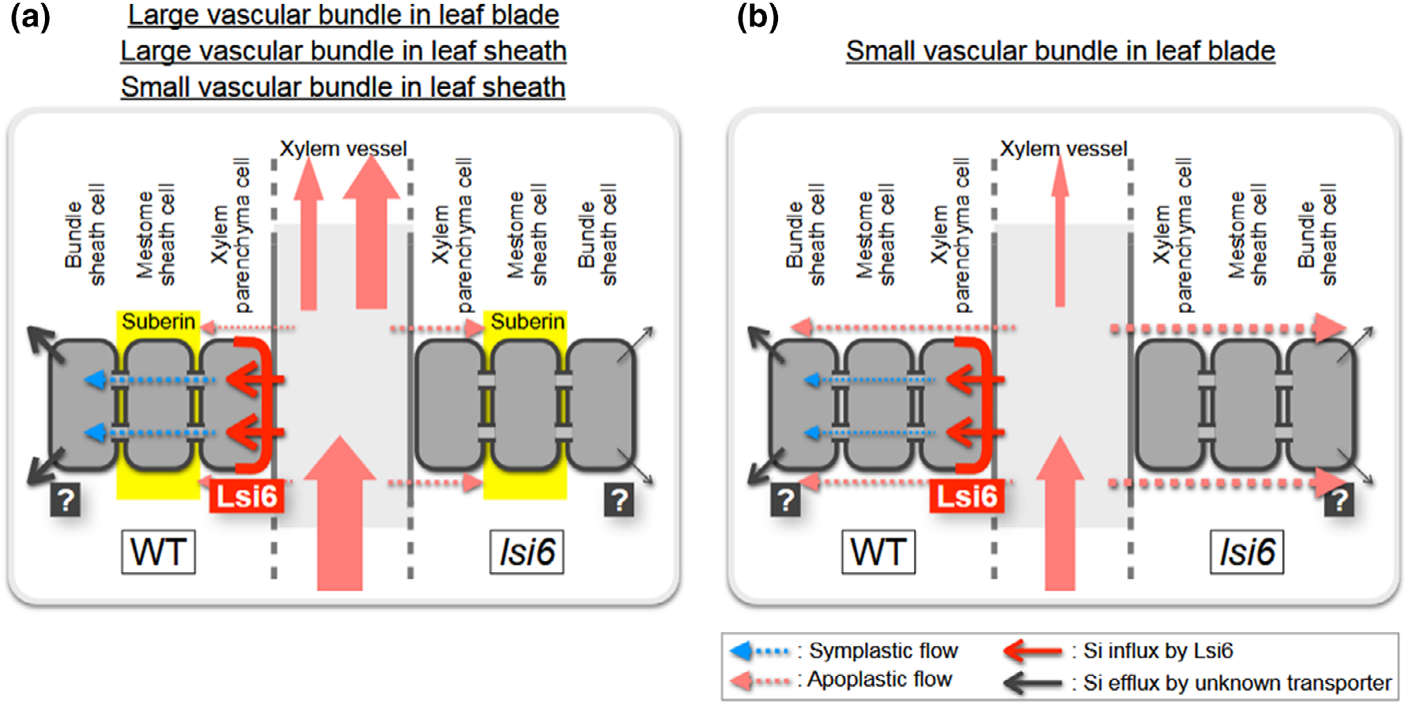
Schematic presentation of the symplastic and apoplastic pathways for the local distribution of silicon (Si) in rice leaves. (a) Symplastic pathway. Due to suberin deposition in the mestome sheath cell layer of large vascular bundles of leaf blade, and the large and small vascular bundles of the leaf sheath, Si is unloaded symplastically by OsLsi6 polarly localized at the xylem parenchyma cells of these tissues and an unidentified efflux transporter. Therefore, the knockout of *OsLsi6* failed to unload Si from the xylem vessels, resulting in higher distribution to the leaf blade and guttation. (b) Apoplastic pathway. Due to the lack of suberin deposition at the mestome sheath cell layer in small vascular bundles of the leaf blade, Si is diffused into leaf tissues through the apoplastic pathway independent of the Lsi6‐mediated symplastic pathway. The thickness of the arrows roughly represents the contribution of each pathway.

In rice leaves, there are two types of longitudinal vascular bundles: large and small vascular bundles based on their size (Hoshikawa, 1989). Large vascular bundles and some parts of small vascular bundles are connected between the leaf sheath and the leaf blade. Each vascular bundle is surrounded by a mestome sheath (inner layer with small cells) and a bundle sheath (outer layer with large cells; Leegood, 2008), although some small vascular bundles in lower leaf blades do not have an entire mestome sheath (Kaneko *et al*., 1980). Therefore, after the release of mineral elements from the xylem vessel, there could be two pathways for further transporting them to other leaf cells (e.g. mesophyll cells); apoplastic pathway and symplastic pathway (Fig. 8). In the small vascular bundle of the leaf blade, the mestome sheath cells were not suberized (Fig. 6); therefore, mineral elements, including Si, could be freely diffused from the xylem to other cells apoplastically without the help of transporters (Fig. 8). This is supported by the permeability of apoplastic dye in leaf blade tissues (Fig. 7). However, in the large and small vascular bundles of the leaf sheath and the large vascular bundle of the leaf blade, the cell wall of the mestome sheath cells was heavily suberized (Fig. 6), forming a physical barrier to prevent apoplastic flow (Fig. 8). Therefore, a transporter is required at the parenchyma cells for the symplastic pathway. Our results show that OsLsi6 is required for this pathway of Si and Ge in the rice leaf sheath.

In addition to its localization at the xylem parenchyma cells of large vascular bundles in the leaves as reported before (Fig. 1; Yamaji *et al*., 2008), we found that OsLsi6 was also localized at the xylem parenchyma cells of small vascular bundles of the leaf sheath and the leaf blade (Fig. 1). Furthermore, similar to the localization at the large vascular bundle, OsLsi6 also shows polar localization in small vascular bundles at the side facing toward the xylem vessel (Fig. 1). This polar localization could facilitate xylem unloading of Si and Ge efficiently and directionally (Fig. 8). This is supported by the finding that the knockout of *OsLsi6* decreased Si and Ge accumulation in the leaf sheath but increased their accumulation in the leaf blade in fully expanded leaves (Figs 3a, 4a). Therefore, in the leaf sheath, Si is first unloaded by OsLsi6 from transpiration flow in the xylem to the parenchyma cells, followed by the symplastic pathway to the mestome sheath (Fig. 8). For further transport of mineral elements out of the mestome sheath cells, an efflux transporter is required (Fig. 8). In the leaf blade, Si is unloaded by OsLsi6 from the transpiration flow in both large and small vascular bundles. On the other hand, Si is also unloaded through the apoplastic pathway in small vascular bundles of the leaf blade (Fig. 8). It was reported that OsBOR1 polarly localized at the proximal side of the mestome sheath cells is responsible for the release of B from the mestome sheath cells (Shao *et al*., 2021), but the transporter for Si release remains to be identified in the future.

In conclusion, our results indicate that OsLsi6 polarly localized at the small and large vascular bundles of the leaf sheath mediates xylem unloading of Si and Ge, but not B and As for their local distribution. We also revealed that there are two pathways for xylem unloading of Si and Ge: the OsLsi6‐mediated symplastic pathway in the leaf sheath and the OsLsi6‐independent apoplastic pathway in the leaf blade based on vascular bundle structure and tissue specificity of OsLsi6 localization. These pathways are likely applicable to other mineral elements, although transporters involved in their xylem unloading remain to be identified in the future.

## Competing interests

None declared.

## Author contributions

SH and JFM conceived and designed the experiments. SH, NY, NM‐U, NK and JFM performed the experiments and analyzed the data. SH and JFM wrote the paper. All authors discussed the results and commented on the manuscript.

## Disclaimer

The New Phytologist Foundation remains neutral with regard to jurisdictional claims in maps and in any institutional affiliations.

## Supporting information


**Fig. S1** Two target sites used for the generation of knockout lines of *OsLsi6* with CRISPR/Cas9.
**Fig. S2** Comparison of micronutrients in leaf sheath and leaf blade between wild‐type rice and *oslsi6* mutants.
**Fig. S3** Transport activity of OsLsi6 for boric acid.
**Table S1** Primer sequences used in this study.
Please note: Wiley is not responsible for the content or functionality of any Supporting Information supplied by the authors. Any queries (other than missing material) should be directed to the *New Phytologist* Central Office.

## Data Availability

The data supporting the findings of this study are available within the manuscript and its Supporting Information (Fig. S1–S3, Table S1).
