# Supplementary material for: Symplastic and apoplastic pathways for local distribution of silicon in rice leaves
Source: New Phytol. 2025 Apr 1;247(3):1280–9. doi: 10.1111/nph.70110 (PMC12222923; doi:10.1111/nph.70110)
Supplement: Supplementary file 1 — Fig. S1 Two target sites used for the generation of knockout lines of OsLsi6 with CRISPR/Cas9. Fig. S2 Comparison of micronutrients in leaf sheath and leaf blade between wild‐type rice and oslsi6 mutants. Fig. S3 Transport activity of OsLsi6 for boric acid. Table S1 Primer sequences used in this study. Please note: Wiley is not responsible for the content or functionality of any Supporting Information supplied by the authors. Any queries (other than missing material) should be directed to the New Phytologist Central Office. [file NPH-247-1280-s001.pdf]

New Phytologist Supporting Information

Symplastic and apoplastic pathways for local distribution of silicon in rice leaves

Sheng Huang, Naoki Yamaji, Noriyuki Konishi, Namiki Mitani-Ueno, and Jian Feng Ma

Acceptance date: 11 March 2025

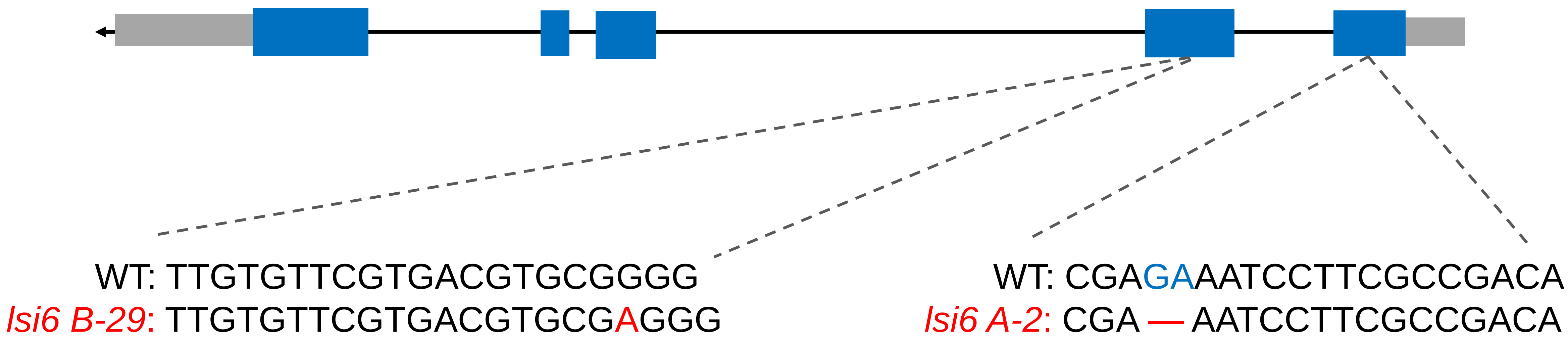

Fig. S1 Two target sites used for generation of knockout lines of OsLsi6 with CRISPR/Cas9. Gray boxes represent UTR regions, blue boxes represent exons, the lines between boxes represent introns.

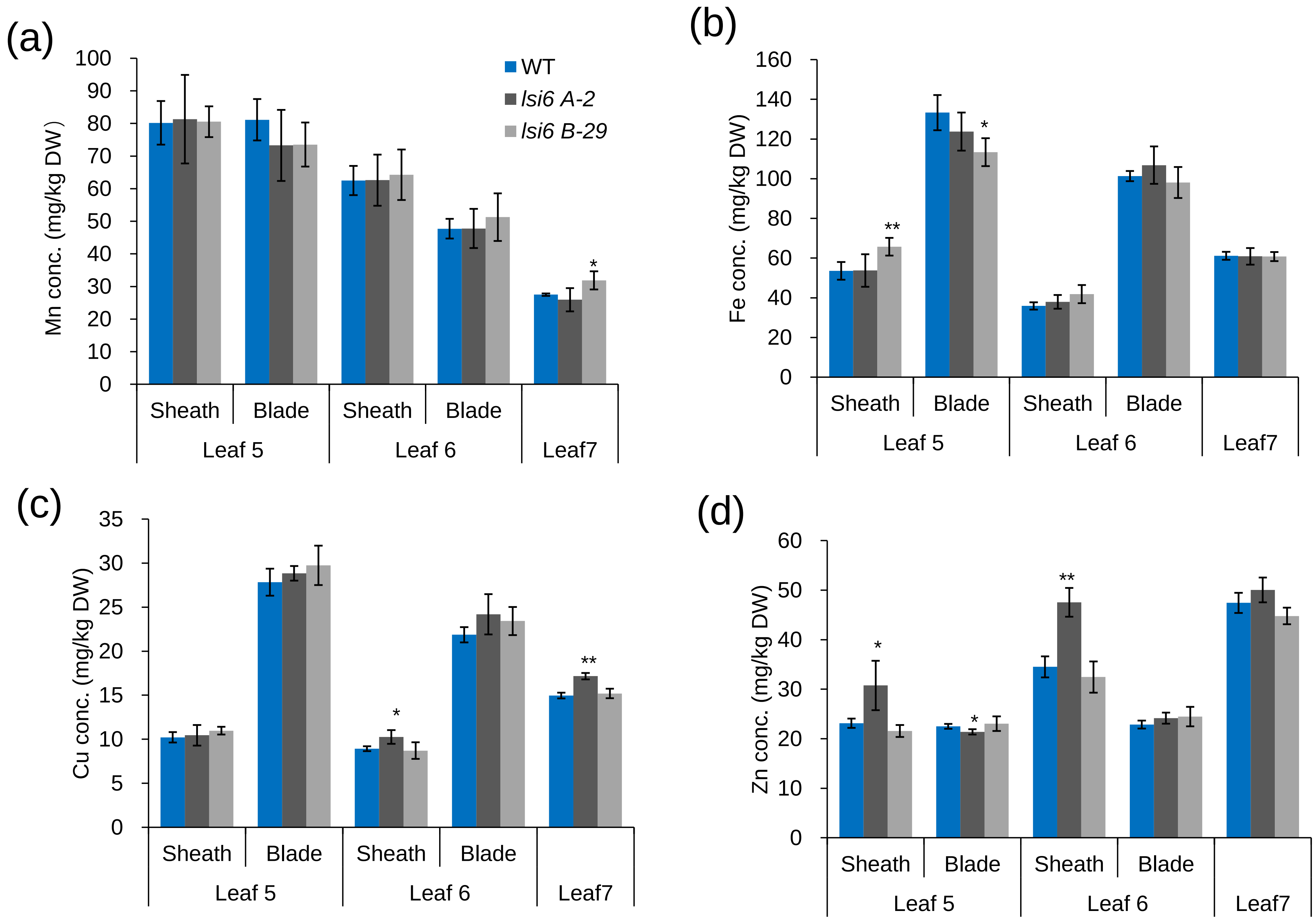

Fig. S2 Comparison of micronutrients in leaf sheath and leaf blade between wild-type rice and *oslsi6* mutants. (a-d) Concentration of Mn (a), Fe (b), Cu (c), and Zn (d) in the leaf sheath and leaf blade of leaf 5 and 6 (fully expanded leaf) and leaf 7 (unexpanded newest leaf). Seedlings of both WT and mutants were grown hydroponically in the presence of 1.0 mM Si for 12 d. The leaf blade and sheath were separately harvested and subjected to the determination mineral elements. Data are means $\pm$ SD (n = 4). Asterisks above bars indicate significant differences compared with WT (*, $P < 0.05$; **, $P < 0.01$), as determined by ANOVA followed by Tukey's test.

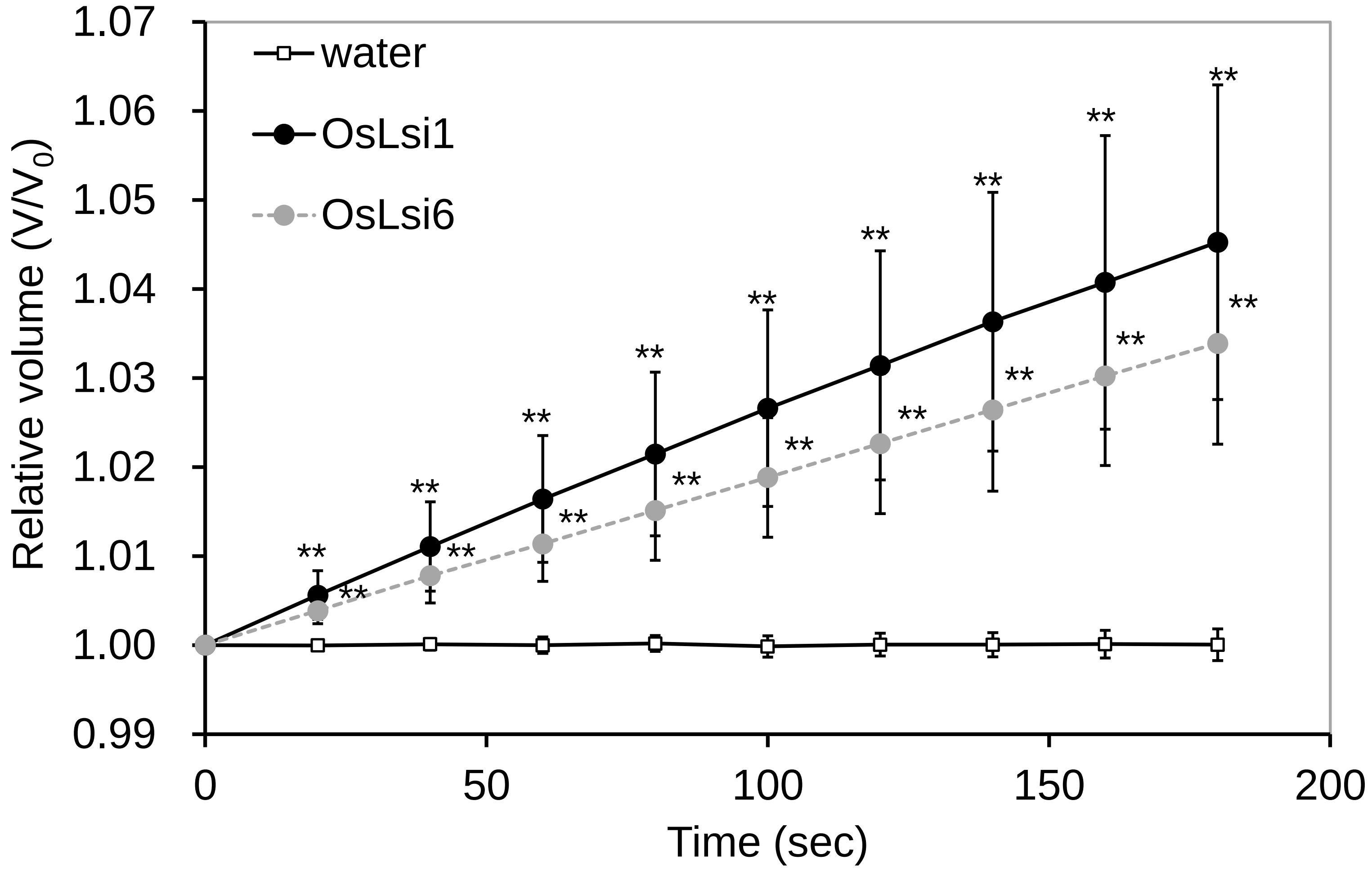

Fig. S3 Transport activity of OsLsi6 for boric acid in *Xenopus* oocytes. Oocytes expressing *OsLsi6* or *OsLsi1* as a positive control were transferred to an isotonic solution containing one-fifth diluted MBS supplemented with boric acid to adjust the osmolarity (boric acid concentration was 175 mM). Changes in the oocyte volume were monitored. Permeability of boric acid was presented as oocyte volume change [V/V_0]. Data are means ± SD (n =10-14). Asterisks above bars indicate significant differences compared with water injection, as determined by ANOVA followed by Tukey's test (**, $P < 0.01$), .

Table S1. Primer sequences used in this study.

Primer name	Forward (5'-3')	Reverse (5'-3')	Purpose
Lsi6-criA	GTTGTGTCGGCGAAGGATTTCTCG	AAACCGAGAAATCCTTCGCCGACA	CRISPR/cas9 system
Lsi6-criB	GTTGTGTGTTCGTGACGTGCGGGG	AAACCCCCGCACGTCACGAACACA	
Lsi6-A-seq	GAGACCAGGGCAAGCAGC	CTGCCGCACACATCATCG	Sequencing
Lsi6-B-seq	CTTCCCTCCTAACCTCCTCAA	CTGAATCCAGGGGAAATGC	
RRT-Lsi6	CGTTGATTCGTTGTCCTAGATAG	GATATCACCTTCTTGAGGAGGTT	qRT-PCR
RRT-Histone H3	GGTCAACTTGTTGATTCCCCTCT	AACCGCAAAATCCAAAGAACG	
RRT-Actin1	GACTCTGGTGATGGTGTCAGC	GTGAGATCACGCCCAGCAAG	
